# Long-term improvement of psoriasis patients’ adherence to topical drugs: testing a patient-supporting intervention delivered by healthcare professionals

**DOI:** 10.1186/s13063-021-05707-6

**Published:** 2021-10-25

**Authors:** Mathias Tiedemann Svendsen, Steven R. Feldman, Sören Möller, Line Planck Kongstad, Klaus Ejner Andersen

**Affiliations:** 1 Department of Clinical Research, University of Southern Denmark, Odense, Denmark; 2 Department of Dermatology and Allergy Centre, Odense University Hospital, Kløvervænget 15, 5000 Odense C, Denmark; 3 Open Patient data Explorative Network (OPEN), Odense University Hospital, Odense, Denmark; 4 Department of Dermatology (Center for Dermatology Research), Wake Forest School of Medicine, Winston-Salem, NC USA; 5 Danish Centre for Health Economics (DaCHE), University of Southern Denmark, Odense, Denmark

**Keywords:** Adherence, Health-care professionals, Psoriasis, Randomized controlled trial (RCT), Support

## Abstract

**Background:**

Psoriasis affects 2–4% of the Western adult population and is a socio-economic burden for patients and society. Topical drugs are recommended as first-line treatment for mild-to-moderate psoriasis, but low adherence is a barrier to treatment success. Psoriasis patients require support, in order to improve their long-term use of topical drugs. The project aims to test whether a patient-supporting intervention delivered by dermatology nurses can reduce the severity of psoriasis, improve the use of topical drugs, and is cost-effective compared to standard procedure.

**Methods:**

The intervention consists of improved support delivered to patients by three experienced dermatology nurses, who will support patients on a regular basis by consultations with a focus on providing reminder systems, accountability, reinforcement, and building trust in the treatment. Each patient will be supported by the same dermatology nurse throughout the entire study period. The effect will be compared with standard procedure.

The intervention will be tested in a randomized controlled trial during a 48-week period. A group of patients with moderate-to-severe psoriasis (psoriasis affecting ≥ 4% of the total body surface area) and 18–85 years of age who are prescribed topical treatment will be randomized to a non-intervention (*n* ≈ 57) or intervention group (*n* ≈ 57). Participants in both arms will be prescribed topical preparations containing corticosteroid and/or calcipotriol.

The primary outcome will be a change in the severity of psoriasis, measured as reduction in the Lattice-System Physician’s Global Assessment. Secondary outcomes will include changes in health-related quality of life (measured by disease specific and generic questionnaires), primary adherence (i.e., proportion of filled prescriptions), and secondary adherence by objective measure (rate of topical drug consumption (obtained by weighing medication packages) compared to estimated recommended consumption). A health economic evaluation is planned to run alongside the trial. Participants’ total health costs will be estimated on the basis of health costs reported to the national health registries and costs spent on the intervention, after which a cost-utility and cost-effectiveness analysis will be carried out.

**Discussion:**

If the intervention can reduce the severity of psoriasis in a significant manner and is economically favorable compared to standard treatment, there is potential for implementing the intervention in dermatology clinics.

**Trial registration:**

Clinicaltrials.govNCT04220554. Registered on January 7, 2020. Study results, either positive, negative, or inconclusive, will be published on www.clinicaltrials.gov.

Trial registration no. with the Danish Regional Committee on Health Research Ethics, registration no. 72613.

**Supplementary Information:**

The online version contains supplementary material available at 10.1186/s13063-021-05707-6.

## Administrative information

Note: the numbers in curly brackets in this protocol refer to Standard Protocol Items: Recommendations for Interventional Trials (SPIRIT) checklist item numbers. The order of the items has been modified to group together similar items (see http://www.equator-network.org/reporting-guidelines/spirit-2013-statement-defining-standard-protocol-items-for-clinical-trials/).

**Table:** 

Title {1}	Long-term Improvement of Psoriasis Patients’ Adherence to Topical Drugs: Testing a Patient-supporting Intervention Delivered by Healthcare Professionals
Trial registration {2a and 2b}.	Trial registration no. on Clinicaltrials.gov: NCT04220554
Protocol version {3}	Protocol version 1.5 dated June 2, 2021.
Funding {4}	Financial donations from: LEO Foundation, Odense University Hospital Free Research Fund, Robert Wehnerts and Kirsten Wehnerts Foundation, Psoriasis Research Foundation, and the Jeweler A.L. Rasmussen Memorial Foundation.
Author details {5a}	MTS conceived the study and wrote the PRO (Patient-reported outcomes)-specific aspects of the trial protocol. SRF and KEA initiated the study design. SM initiated the statistical data analysis plan. LPK initiated the plan for the economic evaluation.
Name and contact information for the trial sponsor {5b}	Klaus Ejner Andersen Department of Clinical Research University of Southern Denmark Winsløwparken 19 Odense C, DK-5000 e-mail: KEAndersen@health.sdu.dk
Role of sponsor {5c}	The sponsor conceived and designed the study, and will participate in the management, analysis, and interpretation of data; the writing of the report; and the decision to submit the report for publication. However, the sponsor will not have ultimate authority over any of these activities. The funders had no role in the study design; data collection, management, analysis, and interpretation; in the writing of the report; or the decision to submit the report for publication.

## Introduction

### Background and rationale {6a}

Psoriasis is a chronic inflammatory skin disease affecting 2–4% of the adult Western population [1]. It is associated with many comorbidities, negatively affects quality of life [2], and is a socio-economic burden for patients and society [3]. Topical drugs are the recommended first-line treatment for mild-to-moderate psoriasis, but patient’s adherence to treatment is low, which is a barrier to treatment success [4]. Poor outcomes result in the need for systemic or biologic treatments that are associated with potential severe adverse events and are often more expensive than topical drugs. Nevertheless, since improved adherence to topical drugs is associated with improved efficacy, there is a need for patient-supporting interventions that improve psoriasis patients’ adherence to topical drugs [5, 6].

Patient support by telephone or smartphone applications (apps) may improve adherence to topical treatment, but the effect is small and has only been studied with a short-term follow-up period [7, 8]. Furthermore, not all patients have access to a smartphone, and technologies may fail to be sufficiently individualized for all patients’ needs.

To improve the effect of topical therapy in psoriasis patients, there is a need for studies on how to optimize the use of available healthcare professionals [9–11]. Since dermatologists are a limited resource, the use of dermatology nurses, who are trained to support psoriasis patients in their use of topical drugs, may be a practical solution for improved health outcomes, which can be measured by an objective reduction in the severity of skin disease and may also be reported by patients in patient-reported outcomes (PROs) (in regard to overall health-related quality of life and dermatology-specific quality of life) [12]. This is supported by clinical trials and focus group studies suggesting that improved support benefits adherence to topical drugs [13–15]. Improved support from dermatology nurses, with specialist training in supporting psoriasis patients may include [1] individualizing treatment plans, [2] providing easy access to dermatology consultation in case of flare-up, and [3] motivating patients to use the medication.

#### Objectives {7}

The study investigates if the use of topical corticosteroids and/or calcipotriol with a patient-supporting intervention delivered by dermatology nurses supports psoriasis patients’ adherence to topical drugs significantly [1] reduces the severity of psoriasis, [2] improves health-related quality of life, [3] improves long-term adherence to prescribed topical drugs, and [4] is cost-effective compared to use of topical corticosteroids and/or calcipotriol with standard patient support.

The null hypothesis is there is no difference in reduction in severity of psoriasis (objectively as measured by the Lattice-System Physician’s Global Assessment [LS-PGA]) among psoriasis patients who receive patient-supporting interventions versus those who do not receive the patient-supporting intervention (the non-intervention group). The purpose of the trial is to test the null hypothesis in a superiority setting.

Furthermore (beside the null hypothesis for the primary outcome), the study will also investigate if there is a difference in quality of life (as measured by the PRO Dermatology Life Quality Index [DLQI]), adherence to medication (as measured by filled prescriptions, amount of medication used and reported by patients in a study-specific questionnaire) and health-care costs among those receiving psoriasis patient-supporting interventions versus those who do not receive patient-supporting interventions (the PRO European Quality of life 5 Dimensions [EQ-5D] questionnaire will be obtained in order to answer this research question).

#### Trial design {8}

The study is a randomized, controlled, investigator-initiated, parallel group with 1:1 allocation ratio, superiority trial (RCT) (Additional file 1) with an intention to treat (ITT) analysis.

## Methods: participants, interventions, and outcomes

### Study setting {9}

The study setting is a single-site study at the outpatient clinic at the Department of Dermatology at Odense University hospital in Denmark. Psoriasis patients who are either already patients at the clinic or newly referred to the clinic will be considered for inclusion.

### Eligibility criteria {10}

All eligible participants will be informed about the study purpose and must consent to participate. Participants will answer questions regarding their socio-demographics and use of medication and will answer questionnaires about their health-related quality of life. Participants are allowed to be in a stable treatment phase with systemic drugs prescribed for dermatological disease.

Inclusion criteria:

- Legally competent patients between 18 and 85 years of age
- Mild to severe plaque psoriasis (psoriasis affecting ≥ 4% of the total body surface area)
- Access to a telephone
- Ability to read the Danish language and basic internet technology skills

During the trial, fertile women need to use a reliable form of contraception, i.e., intrauterine device (IUD), or hormonal contraception (including vaginal ring or birth control injection, implant, transdermal contraceptive patch, or birth-control pill), have a sterile partner, or use dual barriers during the trial period and for at least 14 days after the study ends. Prior to inclusion in the trial, evidence of a negative pregnancy test must be given to the investigator.

Exclusion criteria:

- Patients who cannot read or understand the Danish language
- Breastfeeding or pregnant patients or fertile women who do not use reliable contraception
- Patients who are allergic to all the potential topical drugs that can be prescribed during the trial

Criteria for exclusion during the trial period:

There are six events that will cause removal from the study: 1) withdrawal of consent at the last study visit in week 48, which entails that the data will not be included in the study analysis; 2) a serious adverse event (SAE) related to the intervention or prescribed drugs; 3) for fertile women, the occurrence of pregnancy; 4) change of diagnosis; 5) prescription of systemic antipsoriatic drugs for a dermatological indication during the trial period; and 6) failure to keep several follow-up appointments.

Eligible criteria for individuals who perform the intervention:

Experienced dermatology nurses will perform the intervention (see Additional file 2 for a detailed description of the dermatology nurses hired for the full study period).

### Who will take the informed consent? {26a}

The investigator’s first contact with potential participants will take place in an individual face-to-face consultation prior to the baseline visit. Prior to presenting information about the study, the investigator will inform potential participants of their right to make another appointment at which a companion (lay representative) can be present. Potential participants will be provided with oral and written information about the study. The consent will also grant the authorities (the regional Data Protection Agency and the Committee on Health Research Ethics) the right to monitor and check the patients’ trial-related data obtained in the electronic research data capture and from the patients’ medical chart (Additional files 7 and 8).

The patient information mentions that the study purpose is to investigate the efficacy of topical drugs, indicates that some participants receive additional nurse-support, stipulates the risks and benefits of the study, potential side-effects, treatment plan, precautions, recording of study relevant data and duty of confidentiality, linkage with register data, audit by authorities (the regional Data Protection Agency and the Committee on Health Research Ethics), access for study personnel (the investigator and study nurses) to enter patients’ medical chart and research data tool while the blinded assessor has limited access to the research data tool, compensation and grievance options, finances, voluntary nature of participation, informed consent, and the possibility of withdrawal from the trial. Lastly, the patient will be informed of the availability of treatment if the patient does not wish to participate in the study. Participants shall be made aware of their right to deliberate for 14 days and shall be given the opportunity to have any doubts resolved.

If the patient has decided to participate in the study, the patient in question will date and sign the informed consent and deliver it personally to the investigator. The informed consent is then to be dated and signed by the attesting investigator on the day the patient is informed. The original consent form will be stored in the patient’s case report form (CRF).

### Additional consent provisions for the collection and use of participant data and biological specimens {26b}

The informed consent grants researchers the right to obtain data for research use from the participants medical chart and to link study data to central health registers (for use of medication and other health services) over a 2-year period prior to inclusion in the trial and during the trial period (Additional files 7 and 8).

## Interventions

### Explanation for the choice of comparators {6b}

The non-intervention group receives the standard-of-care treatment for clinical practice at Danish dermatology outpatient clinics. Psoriasis patients are typically seen by a dermatologist at first visit and instructed by a nurse, and every third month thereafter they consult a dermatologist at the outpatient clinic.

#### Intervention description {11a}

##### Intervention group: Detailed description of the improved patient-support

**Fig. 1:**
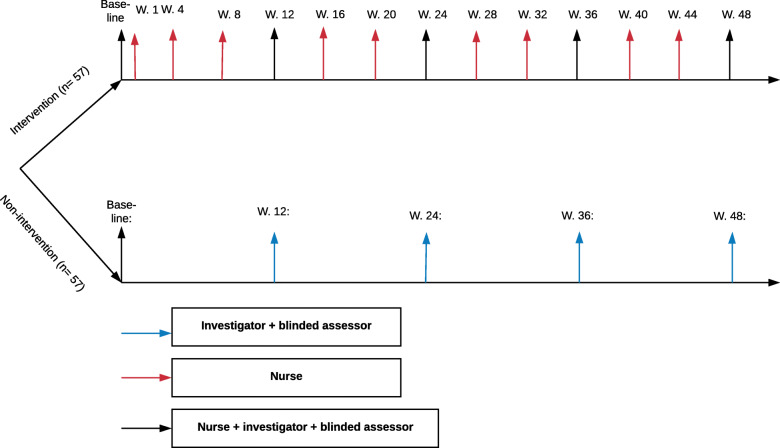
Participants’ visits during the trial period. W, week. The study personnel participating in the patient visits are indicated in the boxes

The intervention group will include support delivered by three experienced dermatology nurses (Additional file 2). Each patient will be supported by the same dermatology nurse throughout the entire study period. The intervention is expected to encourage psoriasis patients to apply the topical drugs on a regular basis by 1) ensuring that patients have a reminder system [16, 17], 2) making patients accountable [18–20], 3) providing support [21], 4) building trust in the treatment and healthcare-provider [10, 13, 22, 23], and 5) by increasing perceived ease of use via favorable comparisons to other treatment options [18, 24] (Table 1). Participants in the intervention group will be seen by a nurse in the dermatology clinic at baseline and weeks 12, 24, 36, and 48 (Fig. 1). Furthermore, the participants will either be seen in the dermatology clinic or receive follow-up from the nurse by telephone at weeks 1, 4, 8, 16, 20, 28, 32, 40, and 44.

**Table 1:** Guide for the patient-supporting consultations. The examples of questions or sentences will be modified by the nurse according to the patient’s needs at the time of the consultation

Theme	Examples of actions, questions, or sentences that the dermatology nurses will use during the consultation
1. **Reminder system**	
Recommending a reminder system	“I really like reminder systems. Most people find a reminder system useful. Are you using one?” If so, “Tell me about your own reminder system.” If not, “what kind of reminders might work well for you?”
2. **Accountability**	
Ask patients to call two days after they have filled their prescription	“Here’s my personal number. Please call me in two days and leave a message telling me what you like and dislike about the treatment”
Ask patients to keep a diary	“I recommend you keep a diary, where you write down any questions and good experiences you have during the treatment.” “I’m looking forward to our next visit/call, to find out how well the medicine is working and to get a sense from you if you've been able to use it regularly. I have a small gift for those of my patients who are able to stick with the treatment plan.”
3. **Reinforcement**	
Giving positive feedback at every follow-up visit	“Your skin looks very good; I can really see that you’re putting a great effort into applying the topical preparations” “I’m impressed to see how well you are in control of handling any flare-ups”
Rewarding patients that fulfill their treatment plans	Give the patient a gift when the patient is half-through the treatment period. The patient will receive some chocolate, for example, with a card specially written for them.
4. **Building trust**	
4.1 Increasing the perceived efficacy of the treatment	
Telling the patient that the prescribed treatment is a good and popular treatment	“I had a patient whose psoriasis was very similar to yours and who did very well on this treatment. Patients really like this treatment”
4.2 Placebo-tailoring (part of building trust in the treatment)	
List many different treatment options, but always recommend one treatment	Have a list of many different treatment options, but always circle the one prescribed for the patient
4.3 Building trust in the healthcare provider	
Optimize the patients' contact with the health care professionals	Hand the patient your personal card. The dermatology nurses write down their telephone number and inform the patient that the number is exclusively for them to use. While writing on the card the dermatology nurse tells the patient: “Here’s my personal telephone number. During the treatment phase if you have any questions about the treatment, please call me and leave a message and I’ll get back to you.”
**5. Increase ease of use via favorable comparison with other treatment options**	
Tell patients about other treatments that have more severe side-effects than the one prescribed	“If you do not use this topical treatment, it might be necessary to prescribe chemotherapy, which has more severe side-effects”
Tell patients that their skin can be treated with a moisturizer once a day, while other patients may need to use many different moisturizers at least four times a day	Tell the patient to use a moisturizer four times a day and then say: “Wait, once a day would be enough for you”
Explain that the treatment regimen is simple	Make the treatment seem simple by saying: “some use this treatment lots of times a day, but now you have learned how to apply the treatment, you can use it once a day”
Make the treatment seem less messy	Tell the patient about the side-effects and messiness using tar and ask the patient if they want to smell a can of tar (have a sample in the consultation room and leave a tar stain on the sink).

##### Intervention group: description of study visits

Baseline visit (expected duration 60 min) (physician 20 min and nurse 40 min): participants are first met by a physician in the dermatology clinic. After obtaining informed consent, patient history and making an objective assessment of the skin, the physician will instruct the patient about topical treatment options and prescribe a topical treatment after shared decision-making [25]. Then, a nurse will instruct the patient on how to use the prescribed topical drugs.

Consultation by nurse in the clinic or digital follow-ups at weeks 1, 4, 8, 16, 20, 28, 32, 40, and 44 (expected duration 20 min) (nurse): participants choose whether they wish to meet the nurse in the clinic or be contacted via telephone, where they may ask questions regarding their treatment and receive further instructions.

Visits at weeks 12, 24, 36, and 48 (expected duration 30 min): participants are seen by the dermatologist and a nurse in the clinic, questions are answered, and adjustment of topical treatment is introduced as needed.

##### Non-intervention group: standard-of-care patient support

The non-intervention group gets the standard-of-care treatment for clinical practice at Danish dermatology outpatient clinics. Psoriasis patients are typically seen by a dermatologist at first visit and instructed by a nurse, and thereafter every third month, they consult a dermatologist at the outpatient clinic.

Visit at baseline and weeks 12, 24, 36, and 48: at baseline, the participant receives 20 min. instruction from the dermatologist and a possible 15 min instruction from the nurse. Subsequent visits consist of a 15 min dermatologist consultation.

##### Study drugs

In both groups, the participants will be prescribed topical drugs based on a shared decision between the prescriber and patient, since preferences for topical drugs differ between patients and there is no single topical drug that is suitable for all psoriasis patients [26]. For both groups, one of the topical drugs containing corticosteroids or calcipotriol registered in Denmark for psoriasis patients will be prescribed (Table 2), since these drugs are the most frequently prescribed topical drugs in Denmark [27] and have a clear dose recommendation [28]. All participants will be instructed how to use the medication according to “the fingertip unit for topical steroids,” i.e., 0.5 g per day per BSA, for as long as a rash is present [28]. No placebo or reference compounds will be used in the trial.

**Table 2:** Topical antipsoriatic drugs that can be prescribed in the study

Drug	ATC class	Type of formulation	Drug class prescription
Clobetasone-17-butyrate	D07AB01	Cream, ointment, cutaneous emulsion, solution or foam	Moderate corticosteroids
Hydrocortisone-17-butyrate	D07AB02		
Betamethasone-17-valerate and betamethasone	D07AC01	Cream, gel, ointment, cutaneous emulsion, solution or foam	Potent corticosteroids
Mometasone furoate	D07AC13		
Fluocinolone acetonide	D07AC04		
Fluocinonide	D07AC08		
Clobetasol propionate	D07AD01	Cream, cutaneous solution, ointment or shampoo	Very potent corticosteroids
Betamethasone and clioquinol, betamethasone and fusidic acid or fluocinolone acetonide and clioquinol	D07BC02	Cream or ointment	Corticosteroids with antimicrobials
Calcipotriol	D05AX02	Cream	Calcipotriol
Betamethasone, calcipotriol	D05AX52	Gel, ointment or foam	Corticosteroid with calcipotriol

*Abbreviation*: *ATC* Anatomical Therapeutic Chemical Classification System

The treatment is not prescribed in accordance with a study protocol, but in accordance with common practice. No extra diagnostic or safety measures will be conducted other than standard care, where the physician informs the patient about common, non-serious as well as rare, serious adverse events and about how to act if these are found.

##### Description and justification of dose level, dose regimen, and frequency plus treatment period

Patients will be treated once daily as long as a flare is present. When calcipotriol-containing topical drugs are prescribed, maximum consumption will be 15 g/day. Since all patients will be prescribed the same group of recommended standard drugs, it is estimated that there are no increased risks connected with participating in the trial.

### Criteria for discontinuing or modifying allocated interventions {11b}

Modifications to allocated interventions will not be permitted.

### Strategies to improve adherence to interventions {11c}

On a weekly basis, the investigator will check if the nurses are providing the planned patient-support and are adhering to the protocol. If the nurses do not adhere to the study protocol, the investigator will immediately contact the study nurse and arrange a meeting, where the study nurse will be instructed about the study intervention guide and asked the reason for non- adherence to the study protocol. Furthermore, participants’ adherence to prescribed medication will be monitored at visit week 48 (see description of adherence measures in section {12}). Visit adherence may be improved by sending SMS reminders to all study participants a few days before each visit. Adherence to treatment is not improved besides the study intervention allocated to the intervention group.

### Relevant concomitant care permitted or prohibited during the trial {11d}

Participants are not allowed to start systemic antipsoriatic drugs prescribed on dermatological indication during the intervention period, since systemic drugs will have an effect on the primary outcome (change in LS-PGA). However, participants who are in a stable phase of prescribed systemic drugs prescribed on dermatological indication at baseline are allowed to be included and kept on the systemic drugs.

### Provisions for post-trial care {30}

Participants discontinued from the study due to the occurrence of an SAE will be followed in the relevant medical department. Participants who participate in the study are covered by the Danish Health Authorities via the Danish Act on the right to Complain and Receive Compensation.

### Outcomes {12}

Outcome measures will be either assessor-blinded LS-PGA and secondary adherence measures of weight of topical drugs used or patient-reported (for patient-reported adherence, DLQ, and EQ-5D) (Table 3). Below, five elements are specified in full for each outcome: (i) domain, (ii) specific measure, (iii) specific metric, (iv) method of aggregation, and (v) time points) [29, 30].

**Table 3:** Enrolment, intervention, and assessment schedule

Study period							
Time points	Enrolment Baseline	Allocation Baseline	Post allocation				
			Baseline	Week 12	Week 24	Week 36	Week 48 (close out)
Enrolment							
Eligibility screen	x						
Informed consent	x						
Allocation: Intervention arm (topical drugs and improved support) or non-intervention arm (topical drugs)		x					
Topical drugs							
Assessments:							
Socio-demographics			x				
Primary outcome:							
LS-PGA			x	x	x	X	x
Secondary outcomes:							
Rates of adherence:							
Primary adherence (proportion of filled new prescriptions)							x
Secondary adherence (by proportion of consumed amount of prescribed medication and in a study-specific PRO questionnaire)							x
DLQI (PRO questionnaire)			x	x	x	X	x
EQ-5D^a^ (PRO questionnaire)			x				x
Total cost of anti-psoriatic treatment			x				x

*Abbreviation*: *DLQI* Dermatology Life Quality Index, *EQ-5D* European Quality of life 5 Dimensions, *LS-PGA* Lattice-System Physician's Global Assessment, *PRO* patient-reported outcome

^a^EQ-5D is collected for use in the health-economy analysis

### Adherence measures

#### Primary adherence

*Specific measure*

Primary adherence will be measured by the proportion of patients who collect their prescribed topical drug (containing corticosteroid or calcipotriol in the trial period within a certain Anatomical Therapeutic Chemical [ATC] class (Table 2)) from a pharmacy during the study period.

*Specific metric*

Value at last patient visit.

*Method of aggregation*

Mean.

*Time point*

Investigated at visit in week 48.

### Secondary adherence

*Specific measure*

Secondary adherence will be calculated by combining measured amount of medication used (determined by the weight of the remains in the used medication packages) per body surface area unit affected.

Estimated recommended consumption of medication will be 0.5 g per day multiplied by the estimated mean BSA during the whole study period, calculated from BSA measures at baseline and at weeks 12, 24, 36, and 48.

$$ \mathrm{Rate}\ \mathrm{of}\ \mathrm{secondary}\ \mathrm{adherence}=\frac{\mathrm{Consumed}\kern0.17em \mathrm{amount}\kern0.17em \mathrm{of}\kern0.17em \mathrm{topical}\kern0.17em \mathrm{drugs}}{\mathrm{Experted}\kern0.17em \mathrm{amount}\kern0.17em \mathrm{used}} $$

Consumed amount of topical drugs = sum of weight of prescribed topical drugs^a^ – sum of weight of all prescribed medication packages returned at visit week 48^b^.

- a: As weighed from representative full medication packages of the prescribed topical drugs.
- b: By weight of medication packages returned by blinded assessor when weighed in the outpatient clinic.

$$ \mathrm{Topical}\ \mathrm{drugs}\ \left(\mathrm{expected}\ \mathrm{amount}\ \mathrm{used}\right)=0.5\;\mathrm{g}/\mathrm{day}\ast 48\;\mathrm{weeks}\ast 7\;\mathrm{days}/\mathrm{week}\ast \frac{\mathrm{BSA}\left(\mathrm{baseline}\right)+2\left(\mathrm{BSA}\left(\mathrm{W}12\right)+\mathrm{BSA}\left(\mathrm{W}24\right)+\mathrm{BSA}\left(\mathrm{W}36\right)\right)+\mathrm{BSA}\left(\mathrm{W}48\right)}{8} $$

*Specific metric*

Value at last patient visit.

*Method of aggregation*

Mean.

*Time point*

The rate of secondary adherence will be assessed at week 48. The measures used to calculate secondary adherence will be obtained as follows: the BSA (1 BSA equals 1% of a person’s total body surface area) will be assessed at each visit (baseline, week 12, 24, 36, and 48) while weight of consumed drugs will be obtained at the last visit in week 48.

### Patient-reported adherence

*Specific measure*

Patients will fill out a study-specific PRO questionnaire, where they report their self-reported adherence (as measured by rate of days where a rash was apparent and the patient applied the prescribed topical preparation (from grades 1 to 4; 1: applied 0–25% of days, 2: applied 26–50% of days, 3: applied 51–75% of days, 4: applied 76–100% of days)). The study-specific adherence measure has previously been used by the research group [8].

*Specific metric*

Value at time-point at last patient visit.

*Time point*

At last study visit at week 48.

## Disease severity measures

### Severity of psoriasis

*Specific measure*

Disease severity will be objectively measured by BSA (an interval scale from 0 to 100; 0, no involvement; 100, full body involvement) and LS-PGA (an ordinal scale from 1 to 8: 1, clear; 8, severely affected) [31].

Disease severity measures will be obtained by a blinded data assessor (a skilled nurse or a qualified trained research assistant). At the appointment the patient is put into a room; the blinded assessor sees the patient first and fills out the scoring (BSA and LS-PGA)—the patient is instructed not to talk except to exchange greetings. The person providing the rating inserts the scoring in the electronic research data capture system.

*Specific metric*

Change from baseline.

*Method of aggregation*

Mean.

*Time point*

Baseline and weeks 12, 24, 36, and 48.

### Dermatology specific quality of life measure

*Specific measure*

The patient fills out a Danish translation of the PRO DLQI questionnaire (an ordinal scale from 0 to 30: 0, not affected by psoriasis; 30, severely affected by psoriasis) [32] questionnaire on a tablet that synchronizes data into the data capturing tool.

*Specific metric*

Change from baseline.

*Method of aggregation*

Mean.

*Time point*

Baseline and weeks 12, 24, 36, and 48.

### Generic health-related quality of life measure

*Specific measure*

The patient fills out a Danish translation of the PRO EQ-5D-3L questionnaire on a tablet, that synchronizes data into the data capture tool. EQ-5D-3L comprises five dimensions: mobility, self-care, usual activities, pain/discomfort, and anxiety/depression. Each dimension has 3 levels: no problems, some problems, and extreme problems. The EQ-5D-3L health profiles are converted into a value based on the Danish societal value sets [33]. The EQ-5D value is on a scale where 1 represents full health and 0 represents being dead; the higher the value, the better the health state. The scale allows negative values to be assigned to health states that are considered worse than dead.

*Specific metric*

Change from baseline.

*Method of aggregation*

Mean.

*Time point*

Baseline and week 48.

### Participant timeline {13}

See Table 3 reporting time schedule of enrolment, interventions, assessments, and visits for participants. The study will be concluded when the last included participant has completed the study.

Regarding PRO assessments, the initial PRO will be collected prior to randomization. PROs will be collected after the clinical assessments. At the visit at baseline and week 48, DLQI questionnaires will be obtained before the EQ-5D questionnaire. At visit week 48, patient-reported adherence will be reported after the EQ-5D questionnaire.

### Study plan and design

At an ordinary consultation with patients referred to the dermatology department, the investigator will screen for suitable participants according to the inclusion criteria. Before patients are included in the study, informed consent will be obtained at the baseline visit.

### Information obtained and provided throughout the study

At the baseline visit, the investigator will collect information from the patient on [1] current and previous use of medicine, [2] length of illness, [3] socio-economic status (marital status, educational level, days of sick leave and income), and [4] severity of disease and disease duration. The information will be used to check that the non-intervention and intervention groups are comparable.

**Fig. 2:**
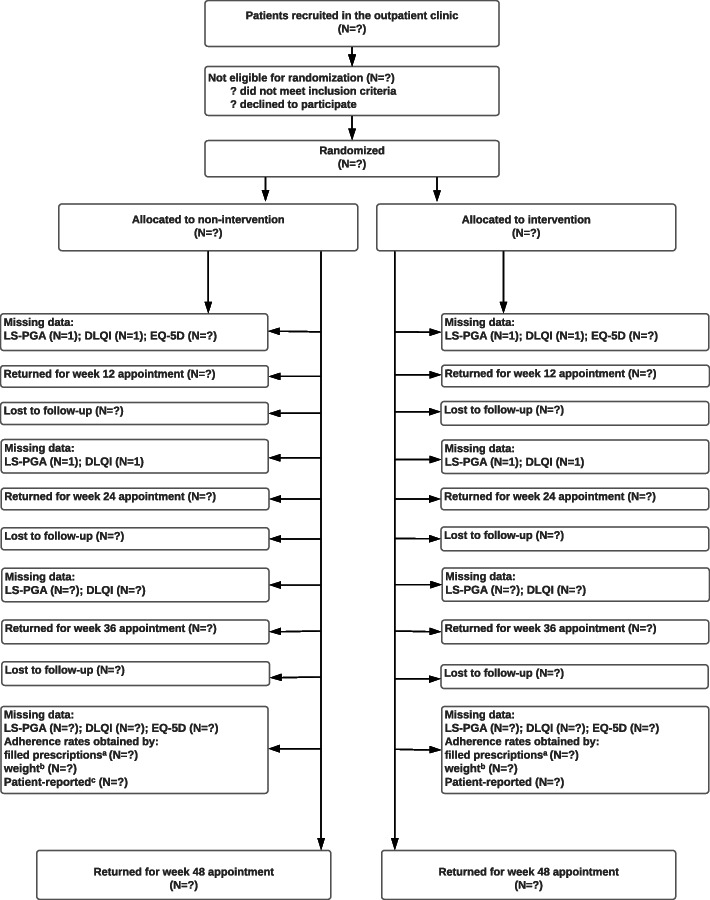
Participant flowchart. Superscript lowercase letter “a” indicates the following: adherence rates obtained according to the proportion of prescriptions filled within 7 days after first prescription. Superscript lowercase letter “b” indicates the following: adherence weight obtained by weight: weight of returned canisters divided by weight of estimated amount of use for the entire study period. Superscript lowercase letter “c” indicates the following: adherence rates reported by patient on a study-specific 4-point interval scale. DLQI, Dermatology Life Quality Index. EQ-5D, European Quality of life 5 Dimensions. LS-PGA, Lattice System Physician’s Global Assessment

The source data that will be stored in Research Electronic Data Capture (REDCap) during the study visit is presented in Fig. 2. In addition, after visit week 48, data from the Danish Health Registries will be obtained in order to have outcomes for primary adherence and costs of health service use.

### Sample size {14}

The null hypothesis is that there is no difference in the primary outcome (LS-PGA) between the intervention and non-intervention groups. The sample size was calculated based on data from a previous project obtaining LS-PGA data from 134 patients randomized to intervention (an adherence-supporting app) or non-intervention (receiving standard-of-care support) [8], where an over 20% LS-PGA improvement in favor of the adherence-support intervention was observed between non-intervention and intervention group. A relative 20% difference in mean scale on the LS-PGA is expected, and a power of 80%, two-sided significance level of 95%, and 1:1 allocation is desirable, considering a maximum expected dropout rate of 44%. Based on mean and standard deviations of LS-PGA from the previous study, this results in a maximum sample size for a two-sided t-test consisting of 115 participants before drop-out. The sample size was calculated using Stata 15 (StataCorp, College Station, TX, USA [34] (Additional file 5).

The sample size is not calculated for the secondary PRO outcomes. However, the principal PRO hypotheses are that the intervention will improve quality of life. A previous RCT conducted by members of our research group [8] had a comparable sample size and was able to detect an improvement in quality of life in favor of the intervention.

### Recruitment {15}

The Dermatology Department will be the primary recruitment and trial site until the maximum planned number of 115 psoriasis patients has been included. Supplemental patient recruitment may be used by contact with dermatologists in practice or by advertisements in local newspapers, at public institutions or social media on the internet.

## Assignment of interventions: allocation

### Sequence generation {16a}

After baseline data has been entered in REDCap, participants will be allocated parallel-assigned 1:1 in random blocks to either the intervention or the non-intervention arm via pre-specified computer-generated block randomization in the REDCap randomization module.

### Concealment mechanism {16b}

The randomization code will be stored by a data manager. Furthermore, participants are informed that the purpose of the study is to investigate the effect of the prescribed topical drugs.

### Implementation {16c}

After informed consent has been obtained, the investigator obtains baseline data from the study participants, which will be entered in the randomization module in the electronic data collection program REDCap. The investigator will assign participants to the intervention based on the results of the randomization.

## Assignment of interventions: blinding

### Who will be blinded {17a}

The data assessor (who measures LS-PGA and the weight of returned medication packages) will be blinded to the allocation.

### Procedure for unblinding if needed {17b}

Participants are not blinded to the intervention. All participants are informed that some receive additional nurse-support while others are followed by the doctor. However, the investigator tells patients the main purpose of the trial is to study use of topical drugs, thus partially concealing the fact that this is an adherence study. Limiting participants’ awareness that the main aim is to study adherence will reduce any positive effect on adherence in the non-intervention group.

## Data collection and management

### Plans for assessment and collection of outcomes {18a}

#### Outcomes

##### Specification and justification of effect parameters

The timeline for the assessment schedule is presented in Table 3. LS-PGA is a validated outcome measure for the estimation of the disease activity of mild-to-moderate psoriasis [35, 36].

DLQI is a licensed validated PRO questionnaire that was first published in 1994 and has been widely used for clinical and research purposes. It has been translated into Danish [37]. The domains include symptoms and feelings, work and school, daily activities, personal relationships, leisure, and treatment. The domains are divided into 10 items. Each item has a score from 0 to 4. The scores are added together and produce a final score from 0 to 30 (0, QOL not affected by skin disease, to 30, QOL severely affected by skin disease). The questions are designed to be completed within a one-week recall period (i.e., last 7 days). It is designed for use in adults, i.e., patients aged 16 years and over. The average completion time of the questionnaire is 2 min, and an electronic version will be used. Usually, no assistance is required. The DLQI is self-explanatory and can be simply handed to the patient who is asked to fill it in. There is no need for detailed explanation. The DLQI will be used in accordance with its user manual. There will be no deviations from its use. The instrument has been reported to have good content and construct validity, and responsiveness in the assessment of psoriasis in adults, and can feasibly be used in clinical trials and practice [37].

EQ-5D is a validated generic licensed questionnaire for quality of life [38]. There are 243 possible health states defined by combining one level from each dimension, ranging from 11111 (full health) to 33333 (worst health). Patients answer how their perceived health is on the day they complete the questionnaire. The EQ-5D-3L questionnaire also include the EQ visual analogue score (EQ VAS). The EQ VAS records patient’s self-rated health on a vertical visual analogue scale, where the endpoints are “Best imaginable health state” and “Worst imaginable health state.” The average completion time of the questionnaire is few minutes, and the questionnaire is cognitively undemanding. A Danish language electronic version will be used. The questionnaire will be used in accordance with the manual and no deviations from its use will be made.

Obtaining LS-PGA and DLQI five times during the study gives extra support to the analysis phase, so a smaller difference can be detected, if this is stable over time.

Primary adherence will be assessed using data from the high-quality Danish National Prescription Registry [39], which is considered valid and reliable. Using package weight to obtain an estimate of secondary adherence is considered acceptable [40]. The patient-reported adherence scale has previously been used in another adherence-trial [8].

All data fields in the REDCap tool are validated: All fields have a minimum and maximum value which helps to narrow down the input options. This means that REDCap prompts an error if a field entry does not meet certain expectations. Furthermore, at end of the trial, all fields will be validated via visual checking by a member of the research staff, in order to ensure accuracy and check that all data are within the prespecified minimum and maximum values. Proxy-reported outcomes will not be used.

The REDCap data collection tool has a built-in function that issues an immediate warning if all fields in the questionnaire have not been completed. At each visit, the investigator will check that no warnings have been issued. Furthermore, PRO assessments will not be taken from participants who discontinue or deviate from the assigned intervention protocol.

##### Data that is considered to be source data

The following source data will be recorded from the patients’ medical chart or from an interview and stored in REDCap [41]: duration of psoriasis, previous and current systemic, biologic, and topical treatment for psoriasis (in a 2-year period prior to inclusion in the study) and socio-demographic data (relationship status, education and job status, municipality, and national origin). During the study, LS-PGA (including BSA) and the weight of consumed topical medication will be assessed by the blinded assessor. DLQI and EQ-5D will be reported by patients. The investigator will obtain data regarding the antipsoriatic drugs prescribed during the study period, and the rate of filled first prescriptions for antipsoriatic drugs during the study period will be obtained. The trial personnel will note down the time used for consultations.

##### Plans to promote participant retention and complete follow-up {18b}

All participants receive an automatically generated notification a week prior to attending in the outpatient clinic. In case of non-attendance, the investigator will contact the participant by telephone and suggest a new consultation.

##### Data management {19}

Study-relevant clinical data will be stored in REDCap hosted at OPEN (Open Patient data Exploratory Network). Furthermore, data obtained in the clinic will be analyzed for missing data and transferred to Scientific Services at the Danish Health Data Authority for use in the economic analysis.

When participants have returned all the medication packages prescribed during the study period at week 48, the medication packages will be weighed by a blinded assessor and returned to study participants.

After the end of the study and after the reports have been submitted and approved by the authorities (the Committee on Health Research Ethics), the sponsor will ensure that the study data are deleted.

##### Confidentiality {27}

All data concerning participant information will be stored in REDCap and will only be accessible for staff members. All collected data will only be traceable by a code.

##### Plans for collection, laboratory evaluation and storage of biological specimens for genetic or molecular analysis in this trial/future use {33}

Not applicable since no biological specimens are used.

## Statistical methods

### Statistical methods for primary and secondary outcomes {20a}

#### Analysis of the primary outcome: changes in LS-PGA

The primary analysis will be carried out on changes in LS-PGA measures from baseline to week 12 and from baseline to weeks 24, 36, and 48 which will be compared between the two groups by linear mixed model for longitudinal data. LS-PGA will be presented in box plots and bar charts.

Two sensitivity analyses on LS-PGA data will be carried out in which all study participants will be included: (i) a complete case analysis and (ii) an imputed analysis (after 100 times multiple imputations by multivariate normal regression on LS-PGA data) with all covariates.

#### Analysis of secondary outcomes: changes in DLQI value and adherence

Changes in DLQI measures from baseline to weeks 12, 24, 36, and 48 will be compared between the two groups by linear mixed model for longitudinal data. DLQI will be presented in box plots and bar charts.

For separate analyses of the three adherence measures (by filled prescriptions, weight of medication and patient reported adherence, respectively), the adherence rate is dichotomized with a selected cutoff of 80%, with adherence rates above 80% considered adherent (a cutoff typically used when studying adherence in chronic diseases) [8, 42]. Dichotomized adherences are compared by using logistic regression.

Statistical analysis will be conducted by an experienced statistician. The statistician will not be blinded to the intervention, as any blinding would be difficult to obtain because the participants in the intervention group are subdivided in three different subgroups (grouped according to the nurse delivering the intervention), while the non-intervention group is kept in one group. However, the analysis will comply with the statistical methods outlined in the study protocol. An interim analysis is not planned.

Obtaining LS-PGA and DLQI five times during the study gives extra strength to the analysis, so a smaller difference can be detected, if this is stable over time.

#### Interim analyses {21b}

No interim analyses are planned, since there are no anticipated problems that are detrimental to the participants, as all topical drugs prescribed in the trial are well-known manufactured drugs.

Criteria for formally halting the trial will be when the last patient visit has taken place.

#### Methods for additional analyses (e.g., subgroup analyses) {20b}

Subgroup analyses of the primary and secondary outcomes by interaction terms in regression models are planned, to investigate if the dermatology nurses or the percentage of nurse-contacts made by telephone affect the intervention.

#### Analysis methods to handle protocol non-adherence and any statistical methods to handle missing data {20c}

Missing data on the primary outcome will be evaluated to assess the occurrence of specific missing data patterns, and if necessary, missing data will be handled by using multiple imputation. Missing effectiveness values are imputed at missing time points using values from available time points. Furthermore, missing data will not be imputed for PRO outcomes or adherence measures.

#### Plans to give access to the full protocol, participant level-data and statistical code {31c}

Access to the full dataset (participant-level dataset and statistical code) obtained in the clinical trial is not planned. The datasets analyzed during the current study are available from the corresponding author on reasonable request. After the end of the study and after the reports have been submitted and approved by the authorities (the Committee on Health Research Ethics), the sponsor will ensure the study data are deleted.

## Supervision and monitoring

### Composition of the coordinating center and trial steering committee {5d}

There is no trial steering committee.

The coordinating center is responsible for overall data management, monitoring and communication at the study site and among the researchers, and general supervision of the conduct of the research project.

The composition of the coordinating center is as follows:

• Principal investigator: MTS

○ Design and conduct of the study

○ Administration of research grants

○ Handling cooperative agreements

○ Drafting contracts in compliance with applicable laws and regulations

○ Publication of study reports

○ Preparation of protocol and revisions and case report forms

○ Recruitment of participants

• Sponsor: KEA

○ Design and conduct of the study

○ Publication of study reports

○ Preparation of protocol and revisions

○ Recruitment of participants

• Three dermatology nurses

○ Supporting the principal investigator during patient consultations and providing the study intervention

• Two blinded assessors

○ Measuring severity of psoriasis (primary outcome) and weighing study medication

### Composition of the data monitoring committee, its role and reporting structure {21a}

To the best of our knowledge, the patient supporting intervention delivered by dermatology nurses in itself does not carry any risks for participants. Therefore, a data monitoring committee is not required. The study is a smaller-size study without critical safety concerns and addresses less-essential outcomes (relief of symptoms and adherence to medication). The Danish Medicines Agency was consulted and concluded the study was not a pharmaceutical trial and, furthermore, a data monitoring committee was not needed to audit the trial.

The principal investigator and sponsor guarantee data and participant safety.

### Adverse event reporting and harms {22}

PRO data will not be monitored during the study for the purposes of providing information about the clinical care of individual trial participants, since patients’ awareness of any fluctuations may impact on the study outcomes.

Screening for AEs will be conducted at baseline and weeks 12, 24, 36, and 48. At each visit, the investigator will systematically screen for all known side effects listed in the product summary for prescribed topical drugs (Table 3) (Additional file 5) both by interviewing the participants, inspecting the skin surface and by reading through all files in the patient chart. The investigator will adhere to instructions for post marketing reporting of adverse drug experiences. Furthermore, a table with all AEs will be published along with study outcomes according to instructions for reporting adverse events (AEs), SAEs, serious adverse reactions (SARs), and suspected unexpected serious adverse reactions (SUSARs). A table summarizing the number of adverse events in the non-intervention vs. intervention group will be used to compare if there are any group-related differences in terms of AEs and to investigate any potential increased use of topical drugs if one of the groups is associated with an increased risk of AEs.

A table summarizing number of adverse events in the non-intervention vs. intervention group will be used to compare if there are any differences between the groups in regard to AEs and to investigate if any increased use of topical drugs in one of the groups is associated with an increased risk of AEs.

If there is a suspicion of a SAE, the study investigator must immediately be contacted, arrange for relevant inquiries, ensure SAE reporting, and assess if the participant should cease participating in the trial.

If participants discontinue the study as a result of an SAE, the following action will be taken: an objective examination of the skin will be undertaken; if hypercalcemia or hypercalciuria is suspected in patients treated with calcipotriol, it must be confirmed by blood test as well as by objective neurological investigation in the neurological department at the nearby university hospital. If the SAE is adrenal suppression or weakening of glycemic control by diabetes mellitus is suspected in patients treated with topical corticosteroids, it must be confirmed by blood test and by an objective investigation in the endocrinology department at the nearby university hospital. If the SAE indicates a suspicion of a cataract or increased intraocular pressure arises in patients treated with topical corticosteroids, this must be confirmed by an objective ophthalmological investigation in the ophthalmology department at the nearby university hospital.

Participants discontinued from the study due to the occurrence of an SAE will be followed in the relevant medical department.

Participants who participate in the study are covered by the Danish Health Authorities via the Danish Act on Complaints and Compensation.

### Frequency and plans for auditing trial conduct {23}

At all times, the investigator will provide direct access to monitoring, auditing, and/or inspection by the Committee on Health Research Ethics, the regional Data Protection Agency, national health authorities, or health authorities from other countries. This auditing will be independent of the investigator and the sponsor. The investigator will allow the authorities (and the Committee on Health Research Ethics) access to monitor and quality check the data. Furthermore, the investigator will provide an annual report to the regional Committee on Health Research Ethics reporting any adverse events occurring during the trial period.

All data used in the analysis will be stored in REDCap, OPEN Analyze, or Scientific Services at The Danish Health Data Authority.

### Plans for communicating important protocol amendments to relevant parties (e.g., trial participants, ethical committees) {25}

Important protocol amendments will be reported to the relevant parties (i.e., the Regional Committees on Health Ethics for Southern Denmark, Denmark, the trial participants, and trial registries) by e-mail. Substantial amendments are only implemented after approval of the Regional Committees on Health Ethics for Southern Denmark, Denmark. All non-substantial amendments are communicated to the Regional Committees on Health Ethics for Southern Denmark, Denmark and within the Annual Safety Report.

### Economic evaluation

To assess the effectiveness of the intervention, a cost-effectiveness/utility analysis will be performed. The effect measures are quality-adjusted life years (QALY), DLQI, and LS-PGA. EQ-5D values (based on Danish societal weights) [33] will be used to calculate QALYs [43]. The intervention has numerous potential short-term economic benefits within the study period, primarily a reduction of the need for prescribing narrow-band ultraviolet B (UVB) treatment, biologic treatment, systemic antipsoriatic drugs, and laboratory analyses used to monitor the use of drugs. Furthermore, the intervention may reduce patients’ need for consulting the general practitioner or private dermatologists to obtain a prescription for antipsoriatic treatment. As the intervention may improve patients’ well-being, there is likely to be a reduced need for psychological or psychiatric treatment. The intervention is not expected to reduce the incidence of other psoriasis co-morbidities, except for improved well-being. Adverse events will be recorded but not included in the economic evaluation model, as they are assumed to be mild for both groups, thus not incurring relevant costs. Costs and resources will be calculated from a healthcare sector perspective including healthcare system costs. The total healthcare costs (measured in GBP in 2021 prices) for participants will be obtained to compare costs for the intervention group and non-intervention. Cost data for the economic evaluation will be compiled by (i) registration at each outpatient visit during the trial period of the time spent by the nurse delivering dermatology support to patients in the intervention group (study personnel will receive a fixed payment per work hour), (ii) registration of cost for drugs delivered free-of-charge (by searching the patient medical charts, estimating drugs linked to a unique pharmaceutical item number (for injection methotrexate and biologic antipsoriatic drugs, list of ATC codes for antipsoriatic drugs provided free of charge can be found in Additional file 3)) [44], and (iii) extraction of data from the Danish Health registries to compile total healthcare costs, linked to participants’ personal identification number, in a 2-year period prior to inclusion in the study and during the study period (48 weeks) (Additional file 3). Data from the Danish Health registries contain real-life pseudonymized individual-level data for study participants. Data will be collected by Scientific Service (Danish: Forskerservice) at The Danish Health Data Authority via joint port access [45] linked with study data using the civil registration number. All analyses will be conducted on Scientific Service’s secure server, using encrypted civil registration numbers. Data will include the total cost of prescription medication (Table 3) (data from The Danish National Prescription Registry [39]) and hospital procedures, including in- and outpatient treatments (data from National Patient Register (LPR (Landspatientregistret)) and the diagnosis-related group (DRG)-grouped National Patient Register [46]). Visits in the primary sector, including those of the general practitioner and practicing dermatologist, will be extracted from the National Health Service Register [47]. In Additional file 3, the selected registers and relevant cost data categories in the registers as well as pharmaceutical number code for drugs delivered free of charge to the study participants are listed (the hospital purchase price will be used in the cost analysis). Over-the-counter medications or compounded drugs will not be included in the analysis.

### Cost-effectiveness analyses

The analysis of resource use and costs will be conducted on individual patients’ aggregated cost measures during the observation period (48 weeks) from the inclusion date for each individual patient. Regression analyses will be used to estimate incremental cost and QALYs. Because costs are normally right-skewed and QALY left-skewed, generalized linear models are considered. As the intervention and non-intervention might have different resource use at baseline, an adjustment is made for baseline costs (cost of the previous 2 years). QALYs are adjusted for baseline utility values [48]. If appropriate, an adjustment for baseline covariates in the regression analysis of QALY will also be applied. Baseline covariates (self-reported) will be age, gender, marital status, education, psoriasis-age, smoking-status, labor market participation, income, and absenteeism. Models are estimated with and without adjustment for covariates. Missing effect data will be evaluated to assess the occurrence of specific missing data patterns and, if necessary, multiple imputation will be used.

The total costs from baseline to 48 weeks after baseline (end of trial) is compared between the two groups and divided by the difference in gain in effect, resulting in an incremental cost-effectiveness ratio (ICER). The ICER is measured as the total cost per QALY gain from baseline to the end of the trial. For this trial, the ICER is a summary measure representing the economic value of the intervention compared to the standard care procedure. A positive ICER reflects the additional costs per one additional unit of the measure of effect. A negative ICER reflects that one of the treatment strategies is dominant. Similarly, an ICER will be computed using DLQI and LS-PGA effect measures. Dominance in the results exists if one strategy is found to be both cheaper and more effective than the other. The clinical relevance of the analysis will be improved if the ICER reflects what is considered a minimal clinically important difference (MCID) in the DLQI and LS-PGA (that is, at least a two-point difference in the DLQI [37] as well as the LS-PGA [36]). Results will be bootstrapped to obtain confidence limits around the estimate [49, 50]. Furthermore, conventional methods to examine the sensitivity of the cost-effectiveness analysis, such as cost-effectiveness acceptability curves (CEAC), will be applied. All analyses will be performed using STATA 16.

### Dissemination plans {31a}

Trial participants and the general population will be informed about the results of the study by means of publication of results on ClinicalTrials.gov. Furthermore, a scientific publication of study results in a peer-reviewed scientific journal is intended.

## Discussion

This study aims to demonstrate whether individualized, optimized patient support (delivered by dermatology nurses) to dermatological patients can optimize the use of topical treatment, reduce the severity of psoriasis, be cost-effective, and have a modest value for improved health outcome compared to standard treatment.

This study is limited by a study sample size calculated on the primary outcome alone (that is, reduction in LS-PGA). Due to the limited sample size, it may be difficult to detect any significant differences between the two groups in terms of secondary outcomes and economic analysis. Since it is felt unlikely for the intervention to have a long-term post-interventional effect on outcomes, the analysis is limited to the intervention period. Data checking will be done by visual checking, which is time-consuming compared to double data entry, for example, but less accurate [51].

The study intervention will be delivered by three dermatology nurses, introducing a risk that the benefits of the intervention are driven (positively or negatively) by a small number of nurses. Development of a detailed study guide, to which the investigator will ensure that study personnel adhere, is a method of reducing the risk that any effect is driven by the dermatology nurses and not the intervention program itself.

The knowledge of participating in an adherence-improving intervention may in itself improve adherence and introduce a risk of reporting bias, in particular when the participants’ use of medication is measured and they report DLQI and EQ-5D. However, it is difficult to blind participants to the intervention given or to obtain approval from the ethics committee for masking participants to the fact that they are in a trial until the end of the study, which has been used in other studies involving adherence-interventions [52].

Patients’ self-reported adherence will be obtained using a study-specific adherence scale, which limits the internal and external validity of the study. It was not considered an option to introduce a placebo-intervention, in order to have blinded patients in the non-intervention arm. This is a limitation of the study design. At the very least, the lack of blinding of the investigator and study nurse introduces a risk of attrition bias while the lack of blinding of the data analyst and health economist introduces a risk of performance bias [53].

A strength of the study is that participants are blinded to one of the facts, namely that a main purpose of the study is to investigate adherence, since the awareness of being in an adherence-improving trial could in itself influence adherence. The study design is based on systematic literature searches and use of a participatory study design, where the opinions of psoriasis patients and dermatology nurses have been considered. The study aims to provide effective, long-term follow-up and to use a clinically relevant primary endpoint (severity of disease), to collect and compare different adherence measures as well as be relevant for potential future implementation of the study (economic analysis). Patient-reported outcomes (DLQI and EQ-5D)) as well as the physician-obtained outcome (LS-PGA) are obtained, which is an advantage of the study since patients’ perception of severity of disease and physicians’ objective measures do not always correlate [54]. To reduce the impact of participants’ awareness of being in an adherence-study, a measure of participants’ estimated adherence is not obtained at baseline (as would have been necessary if, for example, the validated adherence-measure Morisky Medication Adherence Scale 4-item (MMAS-4) was applied at the baseline study visit and at week 48 [55]).

If the intervention is significantly superior compared to standard patient support, there is potential for implementing the patient-support intervention in the dermatology clinic. Results from the study may also be applied to other chronic dermatological diseases. At the very least, the study may be used methodically as a model for research projects investigating other chronic diseases.

## Trial status

### Study schedule

Protocol version 1.5 dated June 2, 2021. The study included its first participant on June 19, 2020. The study is to be completed by summer 2022 at the latest.

## Supplementary Information

**Additional file 1:.** World Health Organization (WHO) Trial Registration Data Set

**Additional file 2:.** List of study nurses’ demographics and experience as dermatology nurses

**Additional file 3:.** Data from patient charts and national registers used for the cost analysis

**Additional file 4:.** Screening and reporting of adverse events (AEs)

**Additional file 5:.** Sample size calculation, Stata script

**Additional file 6:.** Name and address of study site and supporting organizations

**Additional file 7:.** Participant information sheet in English translation

**Additional file 8:.** Informed consent form in English translation

## Abbreviations

AE: Adverse event
ATC: Anatomical Therapeutic Chemical
BSA: Body surface area
CEAC: Cost-effectiveness acceptability curve
CRF: Case report form
DDPA: Danish Data Protection Act
DLQI: Dermatology Life Quality Index
DRG: Diagnosis-related group
EQ-5D: European Quality of life 5 Dimensions
EQ VAS: European Quality Visual Analogue Score
ICER: Incremental cost-effectiveness ratio
ITT: Intention to treat
IUD: Intrauterine device
LPR: Landspatientregistret
LS-PGA: Lattice System Physician’s Global Assessment
MCID: Minimal clinically important difference
MMAS-4: Morisky Medication Adherence Scale 4-item
OPEN: Open Patient data Exploratory Network
PRO: Patient-reported outcome
REDCap: Research Electronic Data Capture
RCT: Randomized controlled trial
SAE: Serious adverse event
SAR: Serious adverse reaction
SPIRIT: Standard Protocol Items: Recommendations for Interventional Trials
SUSAR: Suspected unexpected serious adverse reaction
UVB: Ultraviolet B
WHO: World Health Organization
QALY: Quality-adjusted life years
