# Supplementary material for: Long-term improvement of psoriasis patients’ adherence to topical drugs: testing a patient-supporting intervention delivered by healthcare professionals
Source: Trials. 2021 Oct 25;22:742. doi: 10.1186/s13063-021-05707-6 (PMC8543428; doi:10.1186/s13063-021-05707-6)
Supplement: Supplementary file 1 — Additional file 1:. World Health Organization (WHO) Trial Registration Data Set [file 13063_2021_5707_MOESM1_ESM.docx]

**Additional file 1**: World Health Organization (WHO) Trial Registration Data Set.

| DATA CATEGORY | INFORMATION |
| --- | --- |
| Primary registry and trial identifying number | ClinicalTrials.gov, NCT04220554 |
| Date of registration in primary registry | January 7, 2020 |
| Sources of monetary and material support | LEO Foundation, Odense University Hospital Free Research Fund, Robert Wehnerts and Kirsten Wehnerts Foundation, Danish Psoriasis Association, Jeweler A.L. Rasmussens Memorial Foundation. |
| Primary sponsor | Professor, DMSc Klaus Ejner Andersen, University of Southern Denmark, Department of Clinical Research, e-mail: KEAndersen@health.sdu.dk |
| Secondary sponsor | None |
| Contact for public and scientific queries | Investigator, MD, PhD Mathias Tiedemann Svendsen, University of Southern Denmark, Department of Clinical Research, e-mail: mtsvendsen@health.sdu.dk |
| Public title | Improving Adherence in Topical Treatment of Psoriasis |
| Scientific title | Long-term improvement of psoriasis patients’ adherence to topical drugs: patient supporting intervention delivered by healthcare professionals |
| Countries of recruitment | Denmark |
| Health conditions and problems studied | Medical adherence in topical treatment of psoriasis |
| Intervention | Intervention:  Participants consult health-care professionals at the dermatology clinic on a regular basis, the Psoriasis patients are encouraged to apply the topical drugs by 1) ensuring that patients have a reminder system, 2) making patients accountable, 3) providing reinforcement, 4) building trust in the treatment and healthcare-provider, and 5) increasing perceived ease of use via favourable comparisons to other treatment options.  Non-intervention:  On a quarterly basis, participants are seen at regular follow-ups by the dermatologist at the dermatology clinic. |
| Key inclusion and exclusion criteria | Inclusion:  Legally competent patients of sound mind between 18 and 85 years of age; milder to severe plaque psoriasis (body surface area (BSA) from 4); access to telephone.  Exclusion:  Patients who cannot read or understand Danish language; breastfeeding or pregnant patients or fertile women who do not use reliable contraception; patients who are allergic to all the potential topical drugs that can be prescribed during the trial |
