# Supplementary material for: Long-term improvement of psoriasis patients’ adherence to topical drugs: testing a patient-supporting intervention delivered by healthcare professionals
Source: Trials. 2021 Oct 25;22:742. doi: 10.1186/s13063-021-05707-6 (PMC8543428; doi:10.1186/s13063-021-05707-6)
Supplement: Supplementary file 2 — Additional file 2:. List of study nurses’ demographics and experience as dermatology nurses [file 13063_2021_5707_MOESM2_ESM.docx]

**Additional file** **2**: List of study nurses’ demographics and experience as dermatology nurses

| Study nurse | Age (years) | Sex | Educational level | Experience as a nurse (years) | Experience as a dermatology nurse (years) |
| --- | --- | --- | --- | --- | --- |
| Nurse 1 | 49 | Female | Bachelor | 25 | 8 |
| Nurse 2 | 39 | Female | Bachelor | 14 | 8 |
| Nurse 3 | 53 | Female | Bachelor | 29 | 15 |
