# Supplementary material for: Long-term improvement of psoriasis patients’ adherence to topical drugs: testing a patient-supporting intervention delivered by healthcare professionals
Source: Trials. 2021 Oct 25;22:742. doi: 10.1186/s13063-021-05707-6 (PMC8543428; doi:10.1186/s13063-021-05707-6)
Supplement: Supplementary file 3 — Additional file 3:. Data from patient charts and national registers used for the cost analysis [file 13063_2021_5707_MOESM3_ESM.docx]

**Additional file 3** The data from national registers used to extract data for the Economic evaluation

**Overview of content in the selected Danish national health registries**

| **Danish registers** | **Person-level data content in the registers** |
| --- | --- |
| **The Danish National Health Service Register** | Data source for health care providers in primary health care |
| **The Danish Patient Register (LPR/LPR-PSYK)** | Data source for somatic and psychiatric hospital care |
| **The DRG-grouped National Patient Register** | Data source for somatic hospital care with DRG-groups and tariffs. |
| **The Danish National Prescription Registry** | Data source of all dispensed prescription pharmaceutical drugs sold in Danish community pharmacies. |

Abbreviation: DRG, Diagnosis Related Group; LPR, Landspatientregistret; PSYK, Psychiatry.

**Antipsoriatic medications delivered free of charge to patients**

| **Name of drug** | **ATC-code** |
| --- | --- |
| Adalimumab | L04AB04 |
| Apremilast | L04AA32 |
| Certolizumab | L04AB05 |
| Cyclosporine | L04AD01 |
| Dimethyl fumarate | L04AX07 |
| Etanercept | L04AB01 |
| Golimumab | L04AB06 |
| Guselkumab | L04AC16 |
| Infliximab | L04AB02 |
| Ixekizumab | L04AC13 |
| Leflunomide | L04AA13 |
| Methotrexate | L04AX03 |
| Risankizumab | L04AC18 |
| Secukizumab | L04AC10 |
| Tildrakizumab | L04AC17 |
| Ustekinumab | L04AC05 |

Abbreviation: ATC, Anatomical Therapeutic Chemical Classification System.
