# Supplementary material for: Long-term improvement of psoriasis patients’ adherence to topical drugs: testing a patient-supporting intervention delivered by healthcare professionals
Source: Trials. 2021 Oct 25;22:742. doi: 10.1186/s13063-021-05707-6 (PMC8543428; doi:10.1186/s13063-021-05707-6)
Supplement: Supplementary file 4 — Additional file 4:. Screening and reporting of adverse events (AEs) [file 13063_2021_5707_MOESM4_ESM.docx]

**Additional file 4**: Screening and reporting of adverse events (AEs)

**Local side-effects on the application site (stated from common to ordinary to very rare):**

**For calcipotriol**

Pruritus, skin irritation, burning or stinging sensation, dry skin, erythema, rash, dermatitis, exacerbation of psoriasis, sensitivity to light, hypersensitivity reactions, angioedema, and facial oedema.

**For topical corticosteroids**

Skin atrophy, telangiectasia, striae, folliculitis, hypertrichosis, perioral dermatitis, allergic contact dermatitis, depigmentation of skin and hair, and colloid milium.

**Systemic reactions – severe adverse events (SAE):**

**For calcipotriol:**

Hypercalcemia and hypercalciuria.

**For topical corticosteroids:**

Adrenal suppression, cataracts, infections, lowering of glycaemic control of diabetes mellitus, and increased intraocular pressure.

**Safety evaluation specification and justification of safety parameters and methods and time points for measurement, recording, and analysis of safety parameters**

At visits from baseline during week 12, week 24, week 36 and week 48 of the study, the study investigator will ensure systematic investigation and inquiry into side-effects. The investigator will interview patients and ask if any of the known topical or systemic side-effects has been observed since the last visit, inspect the skin and look through the electronic patient chart. The starting point of this inquiry is the product resume for the respective prescribed topical drugs. Any side-effects that require treatment will be taken care of and followed-up until they are resolved and stabilized. All participants will be informed orally and in writing at baseline that, upon any suspicion of the appearance of a side-effect, they must immediately contact the investigator. Furthermore, all participants will be supplied with a business card with 24-hour direct contact information to a cell phone in the possession of the investigator, who will remain within a maximum 2-hour distance from the trial site during the entire trial period. Since the study is not a pharmaceutical trial, the responsibilities of the investigator, sponsor or trial personnel do not differ from the standard liability that applies for patients not enrolled in the trial. If the study personnel become aware that a study participant has developed a suspected adverse event, the investigator will be informed, and the authorities (the Committee on Health Research Ethics) will be informed immediately if necessary and this will be indicated in the annual safety report. The investigator is responsible for informing The Danish Medicines Agency about potential side-effects.

**Procedures for recording and reporting events and side-effects**

The following terms for events/side-effects will apply in the study: Adverse Events (AE): Any unwanted medical event affecting the participant that does not necessarily have a causal connection to the topical treatment. During the study, all AEs will be observed by the investigator, who will be in charge of recording the AE in the participant’s journal and case report form (CRF). The action taken with the observed AE depends on the causality to the topical treatment.

Adverse Events are classified into ordinary side-effects (cutaneous manifestations) and systemic manifestations (SAE and SUSARs (Suspected Unexpected Serious Adverse Reaction)), see listed side-effects above.

SAEs and SUSARs are systemic side-effects that include one or more of the following points: they 1) are lethal, 2) are life-threatening, 3) require hospitalization (non-elective and of at least one day’s duration) or extended hospitalization, 4) result in permanent or significant invalidity, 5) result in congenital anomaly/birth defect or 6) result in other significant medical events (for example, allergic bronchospasms, cramps or affected blood tests).

All AEs that arise after the participant is included in the study will be recorded in the case report form. The following information will be recorded: 1) description of the event (toxicity and symptoms), 2) time the symptoms debuted, 3) evaluation of the degree of severity, 4) administration of treatment and solution, 5) time the condition stabilized or disappeared, and 6) relationship to the test drug.

**Investigator’s obligations with respect to all Serious Adverse Reactions (SARs) and SUSARs**

SARs and SUSARs will be reported by the investigator to the Committee on Health Research and the Danish Medicines Agency in accordance with applicable legislation

**Causality with respect to AEs, SAEs and SUSARs**

The relationship of the event to the test drug is based on four degrees of causality:

1. Not related: The event is not related if exposure to the test drug has not taken place or it can be ascribed to another cause.

2. Unlikely: The event is not temporally related, or it is not found likely that the event is related to the test drug.

3. Possible: the administration of the test drug and the event are reasonably temporally related, and facts indicate a reasonable causality between the drug and the event.

4. Probable: the administration of the test drug and the event are reasonably temporally related; the event can be related to the test drug more closely than it can be ascribed to other causes or the test drug is highly probably the cause.

Every conceivable medical condition that exists when a participant is screened is considered as baseline without being related to the test drug. If the condition is exacerbated during the study, it will be reported as an AE. The highest degree of an AE will be recorded in the participant’s CRF.

All medically significant AEs that are considered by the investigator or the sponsor to be related to the prescribed topical antipsoriatic drug shall be followed until they have disappeared or are assessed to be stable. Based on the investigator’s clinical decision, the degree of severity of an AE may be assessed to be so pronounced that the participant must discontinue treatment. At any applicable time in the study, participants may express a desire to discontinue treatment because of their own feelings of intolerable toxicity. In both cases, the participant will be encouraged to undertake an “end-of-study assessment” (week 48) and remain under medical observation until the symptoms have ceased or the condition is stable.

**Annual safety report**

Once annually during the study period after inclusion of the first participant, the investigator (the sponsor has delegated this responsibility to the investigator) will prepare a list of all serious putative side-effects that have appeared during the study period and a report on the safety of the participants. The list and report will be signed by the sponsor and sent to the Committee on Health Research Ethics. The deadline for submission of the first safety report (date of confirmation + 60 days and 1 year) will be observed.

All side-effects and events will be reported at the conclusion of the study in the final report to the Committee on Health Research Ethics.
