# Supplementary material for: Long-term improvement of psoriasis patients’ adherence to topical drugs: testing a patient-supporting intervention delivered by healthcare professionals
Source: Trials. 2021 Oct 25;22:742. doi: 10.1186/s13063-021-05707-6 (PMC8543428; doi:10.1186/s13063-021-05707-6)
Supplement: Supplementary file 5 — Additional file 5:. Sample size calculation, Stata script [file 13063_2021_5707_MOESM5_ESM.docx]

**Additional file 5**: Sample size calculation, Stata script

Sample size calculation: Script from Stata 15 (StataCorp, College Station, TX, USA). The sample size is calculated based on a dataset with LS-PGA outcome from research by Svendsen MT et al, published in Svendsen MT. et al. A smartphone application supporting patients with psoriasis improves adherence to topical treatment: a randomized controlled trial. British Journal of Dermatology (2018) 179, pp. 1062-1071.

Sample size calculated based on an estimated 20% difference from LS-PGA baseline.

. di 4.41*1.20

5.292

. power twomeans 4.41 5.29, sd(1.23)

Performing iteration ...

Estimated sample sizes for a two-sample means test

t test assuming sd1 = sd2 = sd

Ho: m2 = m1 versus  Ha: m2 != m1

Study parameters:

        alpha =    0.0500

        power =    0.8000

        delta =    0.8800

           m1 =    4.4100

           m2 =    5.2900

           sd =    1.2300

Estimated sample sizes:

            N =        64

  N per group =        32
