# Supplementary material for: Long-term improvement of psoriasis patients’ adherence to topical drugs: testing a patient-supporting intervention delivered by healthcare professionals
Source: Trials. 2021 Oct 25;22:742. doi: 10.1186/s13063-021-05707-6 (PMC8543428; doi:10.1186/s13063-021-05707-6)
Supplement: Supplementary file 6 — Additional file 6:. Name and address of study site and supporting organizations [file 13063_2021_5707_MOESM6_ESM.docx]

**Additional file 6**: Name and address of study site and supporting organizations

**Unit responsible for the randomization process and statistical analyses of clinical data and providing assistance for storing data**

Open Patient data Explorative Network (OPEN), Odense University Hospital, Odense, Denmark & Department of Clinical Research, University of Southern Denmark, Odense, Denmark, J.B. Winsløws Vej 9 A, DK-5000 Odense C

**Unit responsible for economic evaluation of the intervention**

Danish Centre for Health Economics (DaCHE), University of Southern Denmark, Odense, Denmark, J.B. Winsløwsvej 9B, DK-5000 Odense C

**Unit responsible managing research funding and assisting in financial accounting**

Forskerservice, Odense University Hospital, Odense, Denmark, J.B. Winsløws Vej 4, DK-5000 Odense C
