# Supplementary material for: Long-term improvement of psoriasis patients’ adherence to topical drugs: testing a patient-supporting intervention delivered by healthcare professionals
Source: Trials. 2021 Oct 25;22:742. doi: 10.1186/s13063-021-05707-6 (PMC8543428; doi:10.1186/s13063-021-05707-6)
Supplement: Supplementary file 7 — Additional file 7:. Participant information sheet in English translation [file 13063_2021_5707_MOESM7_ESM.docx]

**Additional file 7:** Informed consent in Danish translation

**Participant information about participating in a scientific experiment.**

Trial title: Long-term improvement of psoriasis patients' adherence to topical drugs: Testing a patient supporting intervention delivered by healthcare professionals

We would like to ask if you would like to take part in a scientific experiment carried out by the person responsible for the experiment, the investigator, specialist in dermatology, PhD Mathias Tiedemann Svendsen and sponsor, professor, DMSc Klaus Ejner Andersen.

Before you decide whether you want to participate in the experiment, you must fully understand what the experiment is about and why we are conducting the experiment. We would therefore ask you to read this participant information thoroughly.

You will be invited to a conversation about the experiment, where this participant information will be elaborated and where you can ask the questions you have about the experiment. Feel free to bring a family member, friend or acquaintance to the interview.

If you decide to participate in the trial, we will ask you to sign a consent form. Remember that you have the right to a reflection period before you decide whether you want to sign the declaration of consent.

Participation in the trial is voluntary. You can withdraw your consent at any time and without giving a reason. It will not have consequences for your further treatment.

**Purpose of the experiment**

In the trial, we want to investigate whether closer follow-up of your psoriasis, which is treated with a prescription topical treatment, can improve treatment results as opposed to standard treatment, where you are followed by check-ups at the dermatologist approximately every third month.

**Method and conduct of the experiment**

In the trial, you will first be seen by a dermatologist who informs you about the trial and obtains informed consent. Then you and the doctor agree on a topical treatment that suits your treatment needs. It is possible that you can be treated with any approved, prescription corticosteroid- or calcipotriol-containing topical treatment that you are instructed to use as needed when there is a flare-up in your skin condition. A lot will be drawn to determine if you have to receive either standard treatment with check-ups at the dermatologist, or additional medical check-ups, approximately every month with a nurse or equivalent healthcare professional. At the patient support consultations with a nurse or support staff, you will talk about how the treatment is going, and you will be instructed and guided in the optimal use of the topical treatment product. The patient support consultations will take place both in the skin department and via telephone conversation, SMS or email.

During the trial, at the doctor's consultations we will obtain data about the severity of your psoriasis. In addition, in the nationwide health registries and your journal, we will collect data regarding other treatments for psoriasis and your consumption of psoriasis drugs for a two-year period, up to the time you were included in the trial, and during the trial period. The purpose of obtaining this information is to investigate whether you have obtained prescription medication and to calculate the total cost of your psoriasis treatment.

Your consent gives the person responsible for the trial, the sponsor and their representatives direct access to health information in your medical record regarding your total consumption of medicines for psoriasis, in order to be able to carry out, monitor and control the trial.

We want to include a maximum of 115 participants in the trial. No biological material will be extracted.

The person responsible for the experiment will ensure that the processing of personal data obtained during the experiment complies with the Data Protection Act and the General data protection legislation, which apply under the Danish data protection legislation.

**Plan for the experiment**

If you participate in the group that receives standard treatment, a total of five visits are planned over 48 weeks. At the visits, the dermatologist will assess the severity of your psoriasis and make adjustments to the topical treatment. If there is a need for additional treatment in addition to the topical treatment, this can be prescribed during the visits. Likewise, during the visits, you can be guided by the skin department's regular nurses. At the last visit in week 48, please bring all empty or half-used tubes of cream from the entire trial period.

If you participate in the intervention group, you will be seen by the dermatologist at five visits over 48 weeks. The dermatologist assesses the severity of your psoriasis and adjusts the topical treatment. At the five doctor visits the visits to a nurse or similar nursing staff will be an immediate extension of the visits to the doctor and will last approximately 20 minutes.

In addition, as part of the planned treatment program, after 1 week and thereafter monthly, follow-up will take place either by appointment, by phone, SMS or email with a nurse or similar non-medical staff, where you are given additional individual guidance and instruction in the use of topical treatment agents.

At the last visit in week 48, please bring all empty or half-used tubes of cream from the entire trial period.

At the end of the trial, we will analyse whether the increased patient support vs. standard treatment is significantly associated with optimized use of topical agents, and if it can reduce the severity of psoriasis, improve quality of life and is cost-effective. We wish to publish the results of the experiment in anonymised form. Depending on the outcome of the trial, we will assess whether there is evidence to introduce the increased patient support as a standard treatment option for all psoriasis patients receiving topical treatment.

**Benefits from participating in the experiment**

You will benefit from being seen by the same dermatologist over an extended period of time and you will receive the best possible topical treatment and the option of other indicated psoriasis treatments offered at the dermatology department of the university hospital. If the trial shows that the optimized patient support reduces the severity of psoriasis, improves the use of medication, improves quality of life and is economically advantageous compared to standard support, there is potential for the optimized patient support to be introduced as a standard offer at dermatological clinics. The trial can be a model for future trials of patient support measures for other chronic diseases.

**Side effects, risks, complications and disadvantages**

Since only registered medicinal products are used in the trial, the trial will not expose you to additional side effects when using corticosteroid- or calcipotriol-containing prescription topical agents. Potential side effects with the use of corticosteroid- or calcipotriol-containing creams, ointments and liniments are listed in **Table 1**. The doctor responsible for the test will systematically ask about any side effects during follow-up visits and ensure follow-up if medication side effects occur.

There may be risks in the experiment that we do not yet know. We therefore ask you to tell us if you experience problems with your health during the trial. Should you experience problems with your health that either relate to the topical treatment that is prescribed to you or to your optimized patient support, please contact the trial manager or the healthcare professional who is responsible for the increased patient support. If we notice side effects or discomfort that we have not already told you about, you will of course be informed immediately and you will have to decide whether you want to continue the trial.

**Table 1**. Potential side effects from topical treatment with corticosteroids or calcipotriol

| Local side effects (from common to very rare) | |
| --- | --- |
| Corticosteroids | Thinning of the skin, rupture of blood vessels, inflammation of the hair follicles, increased hair growth, rashes around the mouth and eyes, allergic contact dermatitis, discoloration of the skin and hair, and milia. |
| Calcipotriol | Itching, skin irritation, burning or stinging sensation, dry skin, redness, rash, eczema, worsening of psoriasis, photosensitivity, hypersensitivity reactions, swelling of the skin or mucous membranes. |
| Systemic possible side effects (all rare) | |
| Corticosteroids | Inhibition of adrenal cortex hormone production, cataracts, infections, inhibited control of diabetes, and increased pressure in the eye. |
| Calcipotriol | Hypercalcemia (elevated calcium in the blood) and hypercalciuria (elevated calcium in the urine). |

As we want results from the trial to be extended to normal clinical everyday life as far as possible, you will not receive free treatment, inconvenience allowance or travel allowance, in addition to what is normally offered for non-trial-related attendance at the hospital.

**Other treatment options**

In the trial, you will have the opportunity to receive other additional psoriasis treatment if the severity of your psoriasis indicates this. The only requirement to continue in the trial will be continued use of topical treatment to a greater or lesser degree.

**Exclusion from and interruption of the trial**

You will be excluded from the trial if you develop an allergy to available topical medications, if new systemic antipsoriasis treatment needs to be prescribed for cutaneous psoriasis, or for women becoming pregnant or breastfeeding. Should the trial show a convincing effect of the patient support as it progresses, completing the trial prematurely will be considered. If the trial is discontinued prematurely, you will be referred for, or instructed on, other relevant treatment.

**Information on financial matters**

The initiative for the experiment was taken by the person responsible for the experiment, investigator Mathias Tiedemann Svendsen.

Trial expenses are financed by grants from the LEO Foundation (grant of DKK 2.2 million), Odense University Hospital's Free Research Foundation (grant of DKK 100,000) and the Robert Wehnert and Kirsten Wehnert Foundation (grant of DKK 25,000). Sponsors have no influence over study design or data collection, analysis or interpretation of experimental findings.

The funding will be used exclusively to pay salary costs for trial staff and to cover trial expenses during the trial period (including recruitment material, printing of trial material and publication and congressional presentation of the trial). The person responsible for the experiment has no financial connection to fund providers.

No consideration will be paid for your participation. You bear your own medical expenses during the trial.

**Access to trial results**

The study is registered on clinaltrials.gov with the registration number NCT04220554. Here, the results of the study, whether positive, negative or inconclusive, will be published. In addition, if the study is successfully completed, an attempt will be made to publish the study results in a peer-reviewed English-language dermatological journal.

The experiment is considered completed by the end of the year 2022. Thereafter, data will be analysed and published.

We hope that with this information you have gained sufficient insight into what it means to participate in the trial and that you feel equipped to make the decision about your possible participation. We also ask you to read the attached material "Subjects' rights in a health science research project".

Processing and storage of personal data takes place in accordance with the Data Protection Act and the General data protection legislation, which apply under the Danish data protection legislation. You can read more here: www.regionsyddanmark.dk/wm513461. If you have questions about the Region of Southern Denmark's processing of your information, you are always welcome to contact the Region of Southern Denmark's data protection adviser via your digital mailbox. These inquiries will be sent encrypted. You can also send an e-mail to databeskyttelsesraadgiver@rsyd.dk. As a rule, these inquiries will not be sent encrypted. Questions about the project should be directed to the principal investigator.

If you want to know more about the experiment, you are very welcome to contact

the principal investigator Mathias Tiedemann Svendsen, e-mail: mtsvendsen@health.sdu.dk.

Yours sincerely

Mathias Tiedemann Svendsen

Principal investigator, specialist in dermato-venereology, PhD.

Department of dermatology and Allergy Center, Odense University Hospital

Kløvervænget 15, DK-5000 Odense C
