# Supplementary material for: Long-term improvement of psoriasis patients’ adherence to topical drugs: testing a patient-supporting intervention delivered by healthcare professionals
Source: Trials. 2021 Oct 25;22:742. doi: 10.1186/s13063-021-05707-6 (PMC8543428; doi:10.1186/s13063-021-05707-6)
Supplement: Supplementary file 8 — Additional file 8:. Informed consent form in English translation [file 13063_2021_5707_MOESM8_ESM.docx]

**Additional file 8**: Informed consent form in English translation

Declaration of consent

The title of the research project: A long-term patient-centred intervention to improve medical

adherence in psoriasis patients topically treated with corticosteroid-containing preparations.

Statement from the subject:

I have received written and oral information and I know sufficient about the purpose, method, benefits and disadvantages to say yes to participating.

I know that it is voluntary to participate and that I can always withdraw my consent without

losing my current or future rights to treatment.

I give consent to participate in the research project, and have received a copy of this consent form as well as a copy of the written information about the project for my own use.

Name of subject: ____________________________________________________________

Date of signature: ____________________________________________

Would you like to be informed about the result of the research project and any consequences for you?

Yes _____ (set x) No _____ (set x)

Statement from the person providing the information:

I declare that the subject has received oral and written information about the experiment.

In my opinion, sufficient information has been provided to enable a decision to be taken on participation in the trial.

The name of the person who provided the information: Mathias Tiedemann Svendsen,

Specialist in dermato-venereology, PhD.

Date Signature: ____________________________________________

Project identification: Registration number at Clinicaltrials.gov: NCT04220554 (registered 7 January 2020), registration number 72613 at the Regional Committees on Health Ethics for Southern Denmark, Denmark (notification submitted 2 March 2020).
